# Improving Polypropylene Matrix Composites Reinforced with Aligned Hemp Fibre Mats Using High Fibre Contents

**DOI:** 10.3390/ma15165587

**Published:** 2022-08-15

**Authors:** Tom Sunny, Kim L. Pickering

**Affiliations:** School of Engineering, University of Waikato, Hamilton 3240, New Zealand

**Keywords:** hemp fibre, natural fibre composites, high fibre content, mechanical properties

## Abstract

The main goal of this study was to improve the mechanical performance of polypropylene (PP) matrix composites through high hemp fibre content. In order to achieve high fibre content, the possibilities of different polymer sheet thicknesses and stacking arrangements were investigated. It was found that decreasing the overall thickness of fibre mats between two polymer sheets within the stacking arrangements of composites and so decreasing the distance the polymer needs to travel improved the fibre wetting and therefore improved the tensile properties. The strongest composite produced had a fibre content of about 60 wt%. At this fibre content, tensile strength and Young’s modulus of the composites were found to be 3.0 and 6.9 times, respectively, higher than the control samples (polymer only samples), while figures for flexural strength and flexural modulus were 3.4 and 3.6, respectively.

## 1. Introduction

In response to the demand of various manufacturing industries for low density and high-performance structural materials at low cost, different composite materials have been developed [1]. Today, many industries, particularly aerospace, sports, and automotive industries, are quite dependent on fibre-reinforced polymer composites [2]. The main potential advantage of polymer matrix composites (PMCs) over conventional structural materials is their low density, which results in higher specific properties of these composites when strong and stiff fibres are used. 

The reinforcement fibres in PMCs can be either synthetic (human-made) or natural and are often encapsulated in a more ductile matrix material. Fibres such as carbon, aramid, and glass are the most commonly used synthetic fibres, with carbon and aramid used in composites that require high performance with extremely high tensile properties. However, the most used synthetic fibres in composites are glass fibres due to their lower cost and acceptable tensile properties. Natural fibres such as hemp, flax, jute, and kenaf are the commonly used fibres in composites [3]. In order to obtain an environmentally friendly composite, the selection of the matrix is also important with biodegradability or recyclability as considerations.

The reinforcing fibres used in composites are generally in continuous or discontinuous forms. The discontinuous fibre composites are becoming more attractive with major benefits, including their low cost and ease of manufacture [4,5]. Previous research outcomes suggest that the best mechanical properties are generally exhibited by the composites when the fibres are aligned to the loading direction [6,7,8]. Various forms of discontinuous natural fibres are available including randomly aligned mats, long yarns, braiding, and woven textiles [9], but literature reports that generally randomly aligned mats are used as reinforcement in natural fibre composites (NFCs) as they are cheap compared with other forms [10]. However, work carried out at the University of Waikato proved that the dynamic sheet forming method is a potential technique to produce aligned short fibre mats [11]. In natural fibre composites (NFCs), the strength of the interface formed has a large influence on composite properties which depends on the mechanism and amount of interaction [12]. The mechanisms of interfacial bonding can be mechanical interlocking (rough fibre surface), chemical bonding (presence of chemical functional groups), and inter-diffusion bonding (interaction between atoms and molecules). There are possibilities of multiple bonding mechanisms occurring at an interface at the same time [13]. The interface strength also depends on the density of bonds [14]. 

Generally, the strength and stiffness of fibre composites are expected to increase with increased fibre content [6], provided these composites have reasonable interfacial bonding between matrix and fibres, as the fibres are usually stronger and stiffer than the matrix. However, research outputs suggest that attempts to increase fibre content above 40–50 wt% generally resulted in declines in tensile properties of the NFCs due to insufficient wetting of fibres by the matrix material [15,16]. 

In the present research, to achieve high fibre content in composites, the effect of different polymer sheet thicknesses and stacking arrangements were investigated. Initially, the stacking arrangements of polymer sheets and fibre mats used for composites were according to the literature [17,18]. Although these arrangements successfully produced composites up to 30 wt% fibre contents, the production was not successful above 30 wt% due to insufficient fibre wetting. It is well known that, for good interfacial bonding to occur, the matrix should be impregnated fully into the space between the fibres and wet fibres. Therefore, in this research, decreasing the overall thickness of fibre mats between two polymer sheets within the stacking arrangements of composites and so decreasing the distance the polymer needs to travel is expected to improve fibre wetting through the composite and therefore improve the mechanical properties of the composites.

## 2. Materials and Methods

### 2.1. Materials

The hemp fibres that underwent high-temperature alkali treatment [19] were used to produce fibre mats. The aligned fibre mats were produced using dynamic sheet forming (DSF). The details can be found in a previously published work [20]. The matrix was polypropylene random copolymer SKRX3600 supplied by Clariant (Rosedale, New Zealand) Limited, with a melt index of 18 g/10 min and with a density of 0.9 g/cm^3^. The coupling agent used was A-C 950P maleic anhydride polypropylene (MAPP) supplied by Honeywell International Inc., Charlotte, NC, USA.

### 2.2. Production of PP/MAPP Sheets

**Figure 1:**
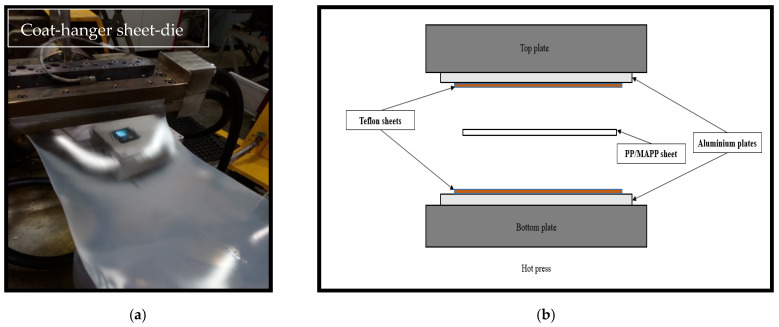
(**a**) The PP/MAPP sheet extrusion using a Labtech twin-screw extruder. (**b**) Schematic representation of reduction of sheet thickness in a hot press.

PP blended with MAPP (PP/MAPP) were formed into sheets using Thermo Prism TSE-16-TC (Waltham, MA, USA) and Labtech 1201-LTE20-44 (Samut Prakan, Thailand) twin-screw extruders attached with a sheet-die (Figure 1a). The former extruder was equipped with a die spacer of 1.00 mm, whereas the latter had a die spacer of 0.50 mm allowing the production of thinner sheets. The heating zones of the extruder barrels were set between 145 °C (feed entrance) and 170 °C (at exit). The rotating screw speed was set to 45 rpm. 

**Figure 2:**
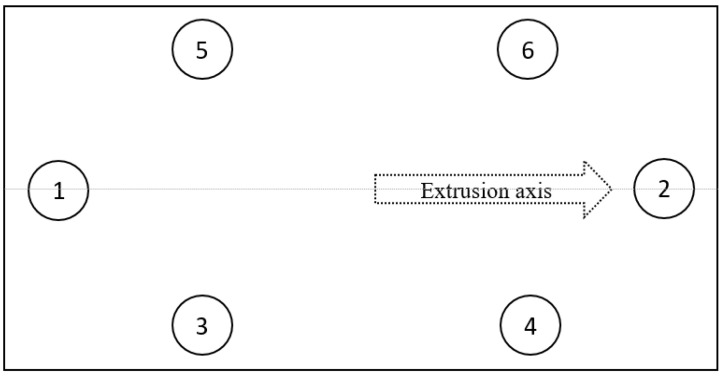
Schematic diagram showing different locations from where the measurements were taken out to obtain the average thickness of the sheets.

To further reduce the thickness of sheets produced with a die spacer of 0.50 mm, they were pressed between two aluminium plates inside a hot press (Figure 1b). The two aluminium plates were lined with Teflon^®^ sheets to avoid polymer sheets adhering to the plates. Based on trial runs, the time (5 min) and applied pressure (1.5 MPa) were maintained as constants with temperatures 130, 140, or 150 °C changed to adjust the thickness of the sheets. After cooling down to room temperature, the sheets produced were cut to the size of the mould used to produce composites. The thicknesses of these sheets were measured at six different points, as shown in Figure 2, using a Vernier calliper. The sheets were then stored in sealed bags for further processing.

### 2.3. Fabrication of Composites 

A series of composites were produced between 30 and 70 wt% fibre contents. To produce these composites, the polymer sheets (PP/MAPP) and the fibre mats were weighed and arranged in stacks between two Teflon sheets (to prevent adhering to the mould). Table 1 shows the stacking arrangements of the polymer sheets and fibre mats with relative numbers of each based on the targeted weight percentage of fibre mats. Stacks were heated and pressed in a hot press same as that of PP/MAPP samples (at 170 °C for 5 min at 1 MPa). Since the fibre mats was easily distorted, the consolidation of PP/MAPP sheets with the fibre mats should be carried out carefully. It was ensured that PP/MAPP sheets were fully melted before slowly applying pressure. After hot pressing, the mould was removed from the hot press and allowed to cool down to room temperature with a weight on the top of the mould (a steel block of 10 kg). Composite samples were then taken out and weighed. The final fibre weight percentage was determined from knowing the weight of fibre mats placed in the mould. All samples were then stored in sealed polyethylene bags. 

A slight modification in the hot-pressing procedure was necessary to produce composites above 50 wt%. The composites were further pressed in the hot press. It has been previously reported that pressing twice could reduce the fibre spring-back in the composite; spring-back is a condition where material partially returns to its original shape due to elastic recovery [21]. This condition often occurs in compression-moulded components when the compaction pressure is released suddenly. To account for this, pressing was repeated. After pressing for the first time, composites were allowed to cool under pressure and then pressed a second time at 170 °C for 5 min at a pressure of 1 MPa. 

**Figure 3:**
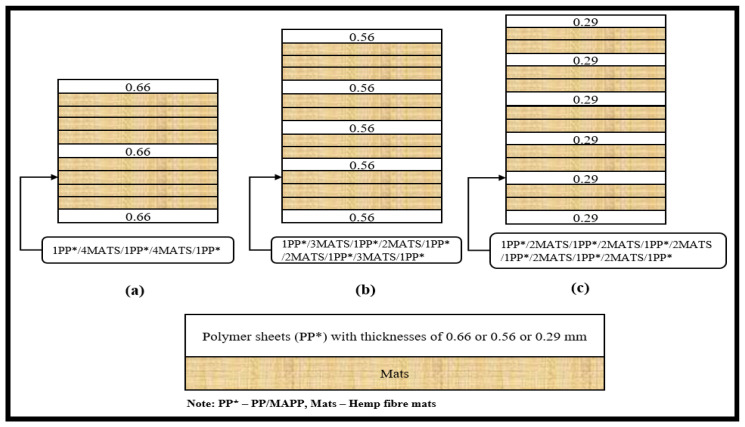
(**a**–**c**) Schematic diagram representing various stacking arrangements used to produce composites with 40 wt% fibre contents. The arrangements of fibre mats and the polymer sheets depend on the thickness of the sheets used.

The possible stacking arrangements of composites were determined by the thickness of polymer sheets and fibre content. For instance, Figure 3 shows the various stacking arrangements used to produce composites with 40 wt% fibre contents. When polymer sheets with an average thickness of 0.66 mm were used, the stacking arrangements consisted of a maximum of four fibre mats between two polymer sheets (left, Figure 3a). When polymer sheets with an average thickness of 0.56 mm were used, it was possible to decrease the number of fibre mats to a maximum of three between two polymer sheets (middle, Figure 3b). When polymer sheets with an average thickness of 0.29 mm were used, it was possible to further decrease the number of fibre mats between two polymer sheets from a maximum of three to two (right, Figure 3c). This stacking pattern of two fibre mats between two polymer sheets was repeated depending on the fibre content and produced composites up to 70 wt%. 

### 2.4. Composite Tensile Testing

Procedures detailed in ASTM D 638-03; Standard Test Method for Tensile Properties of Plastics was followed for testing the specimens [22]. In advance of tensile testing, all the samples were conditioned at 23 ± 3 °C and 50 ± 5% relative humidity for at least 48 h. An Instron-4204 tensile testing machine fitted with a 5 kN load cell was used for the testing (Norwood, MA, USA). The gauge length of the specimens was 80 mm. An Instron 2630-112 extensometer with a gauge length of 50 mm was attached to the central part of the test specimen for the measurement of strain. The specimens were tested at a crosshead speed of 1 mm/min. A total of five samples were tested from each batch. The specimens without fibres were tested only to maximum stress point because of excessive necking, whereas specimens with fibres were tested to failure [18]. 

### 2.5. Assessment of Composite Morphology

Tensile fracture surfaces of composites were investigated using a Hitachi S4100 field emission scanning electron microscope (Tokyo, Japan). 

### 2.6. Composite Flexural Testing

Flexural testing (three-point bending) was performed on an Instron-4204 fitted with a 5 kN load cell according to the ASTM D 790-3 (Standard Test Methods for Flexural Properties of Unreinforced and Reinforced Plastics and Electrical Insulating Materials) [23]. The crosshead speed and unsupported spans were 1.5 mm/min and 48 mm, respectively. Five test specimens with dimensions of 75 mm× 12.7 mm× 3 mm were tested for each batch of samples. The average flexural properties were calculated.

### 2.7. Composite Impact Testing

Impact testing was carried out according to the EN ISO 179-1 Plastics Determination of Charpy Impact strength [24]. An advanced universal pendulum impact tester (POLYTEST) with an impact velocity of 2.9 m/s and a hammer weight of 0.475 kg at 22 °C was used. Five test specimens with dimensions of 75 × 10 × 3 mm were tested for each batch of samples. 

## 3. Results and Discussion

### 3.1. Production of PP/MAPP Sheets

**Figure 4:**
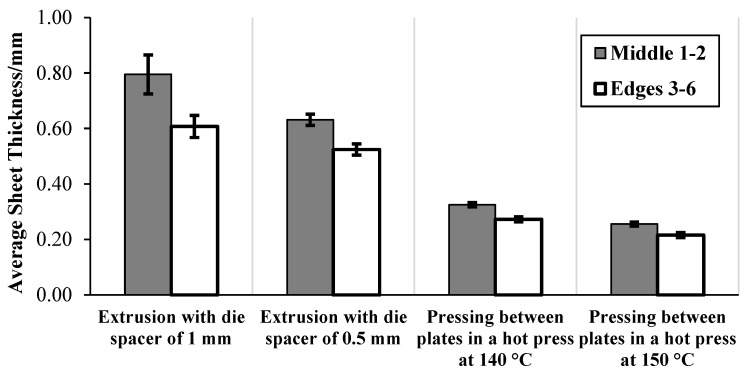
Graph displaying the average thicknesses of the sheets along the extrusion axis 1–2 (middle 1–2) and at the edges 3–6.

As aforementioned, the thickness of each sheet was assessed at six different points. The extruded sheets were slightly thicker (about 23%) in the middle than at the edges (Figure 4). This could be due to the melt flow pattern of the material through the sheet die [25]. In contrast, after pressing between the aluminium plates, the sheets appeared to be slightly more uniform (about 18% difference only between the middle and the edges), with small standard deviations observed, as indicated by the error bars.

Table 2 details the overall average thickness of the polymer sheets calculated from the six different points. As expected, after pressing in the hot press, the overall average thickness of the sheets (produced with a die-spacer of 0.50 mm) reduced, although not significantly for 130 °C (confirmed by the Student’s *t*-test). At 160 °C, due to reduced viscosity, holes over ~40% were formed within the sheets produced, making these unusable in composites.

### 3.2. Tensile Properties of Composites

**Figure 5:**
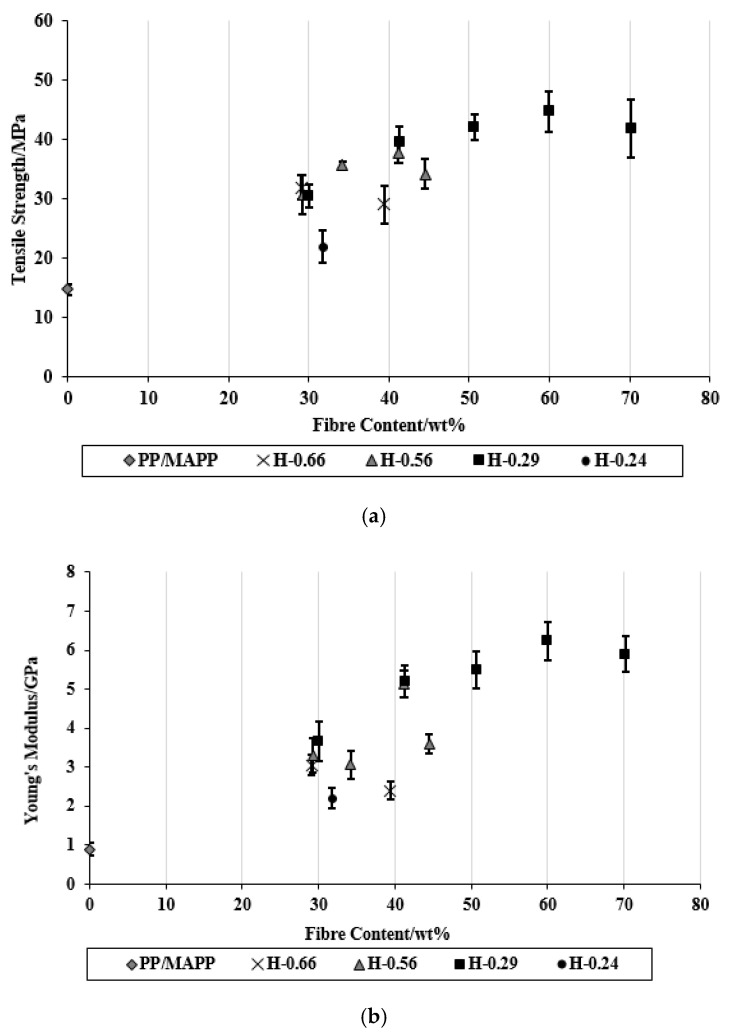
(**a**) Tensile strength and (**b**) Young’s modulus of composites as a function of fibre content.

**Figure 6:**
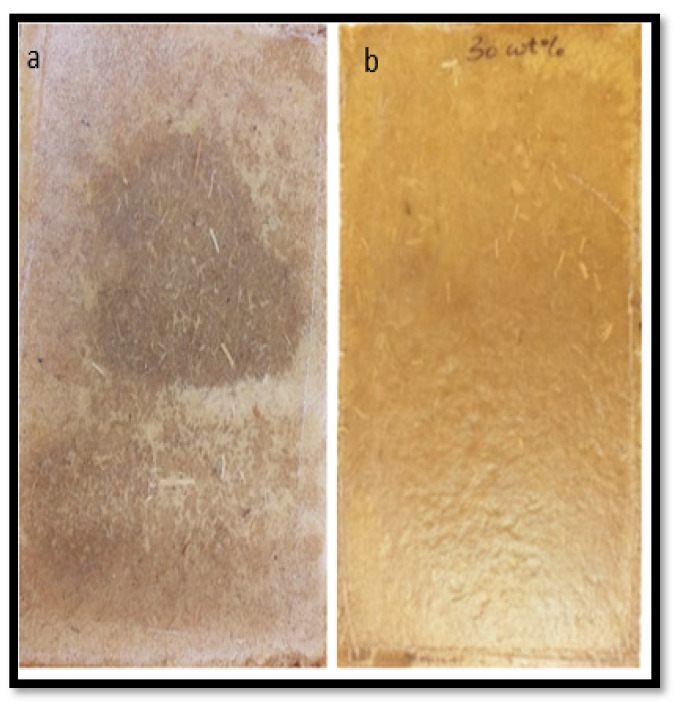
Example photographs of the composite surfaces made with polymer sheets of: (**a**) 0.24 and (**b**) 0.29 mm.

**Figure 7:**
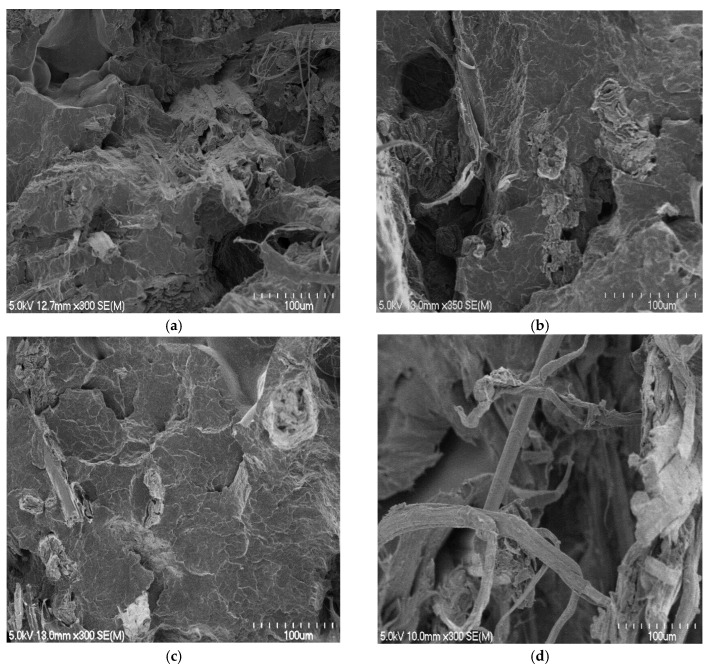
Sample scanning electron micrographs of tensile fracture surfaces of composites containing approximately 30 wt% fibre contents made with polymer sheets of: (**a**) 0.66, (**b**) 0.56, (**c**) 0.29, and (**d**) 0.24 mm.

In this investigation, a range of composites was produced using different sheet thicknesses. Generally, it was found that decreasing the overall thickness of fibre mats between two polymer sheets within the stacking arrangements of composites and so decreasing the distance the polymer needs to travel improved the fibre wetting through the composite and therefore improved the tensile strength and Young’s modulus of the composites (Figure 5). However, the composites made with polymer sheets of 0.24 mm had the lowest tensile strength and Young’s modulus. Although these composites had the least distance the polymer needs to travel (equal with the distance the polymer needs to travel in composites made with polymer sheets of 0.29 mm), the polymer content at the edges was found to be lower compared with other composites, as indicated by the lowest ratio of thicknesses of polymer sheets to the fibre mats at the edges (see Table 3) and hence resulted in a weaker composite. It appears from the photographs of these composite surfaces (Figure 6, composite at the left compared with the one at the right) that the polymer content at the edges was insufficient to bring about reasonable fibre wetting. The SEM micrographs of the tensile fracture surfaces of these composites (Figure 7) also indicated poor fibre wetting; there appeared to be large protruding fibres with no evidence of matrix material adhering to their surfaces, unlike other composites in which very few fibres can be observed as the fibres are mostly covered by the matrix material, suggesting reasonable fibre wetting. For composites containing approximately same fibre content (30 wt%), the use of different sheet thicknesses did not result in significant differences in composite tensile strength and Young’s modulus, except for composites made with polymer sheets of 0.24 mm.

**Figure 8:**
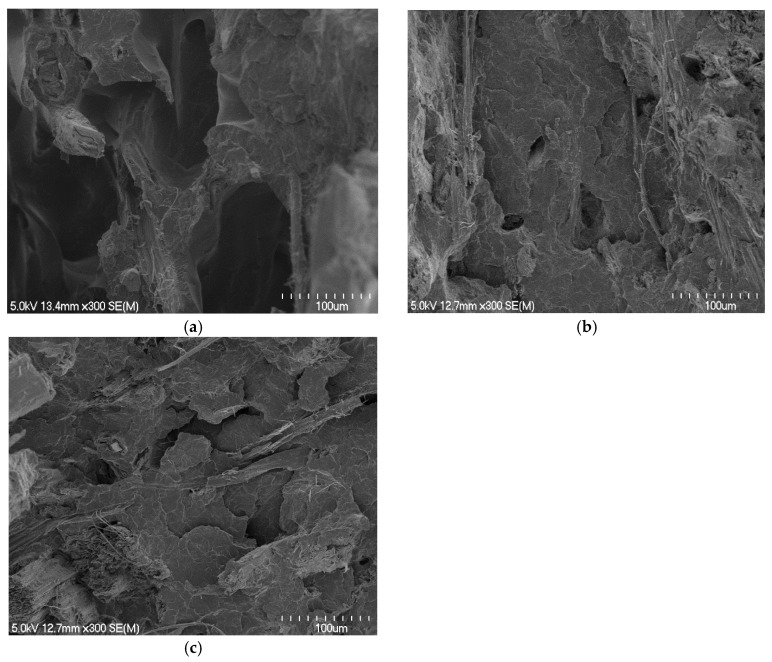
Scanning electron micrographs of tensile fracture surfaces of composites containing approximately 40 wt% fibre contents made with polymer sheets of: (**a**) 0.66, (**b**) 0.56, and (**c**) 0.29 mm.

**Figure 9:**
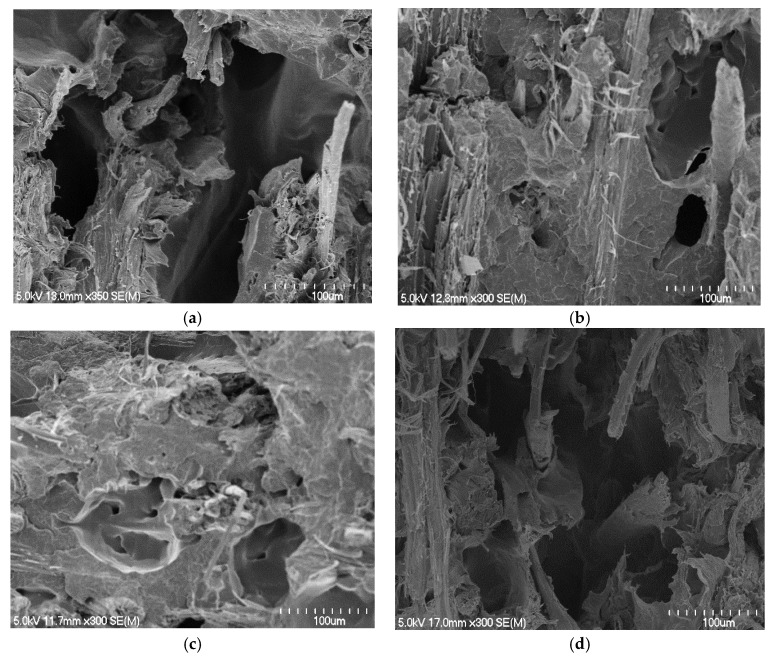
Scanning electron micrographs of tensile fracture surfaces of composites containing fibre content of (**a**) 45 wt% made with polymer sheets of 0.56 mm, (**b**–**d**) 50, 60, and 70 wt% made with polymer sheets of sheet thickness of 0.29 mm, respectively.

Composites made with polymer sheets of 0.66 mm showed significant decreases in the tensile properties when the fibre content was increased from 30 to 40 wt%, unlike other composites made with polymer sheets of 0.56 or 0.29 mm, which showed significant increases. The use of thinner polymer sheets (0.66 compared with 0.56 or 0.29 mm) made possible decreases in the overall thickness of fibre mats between two polymer sheets within the stacking arrangements of the composites. This reduced the distance the polymer needs to travel and improved the fibre wetting. The tensile fracture surfaces of composites made with polymer sheets of 0.66 mm appeared to have large holes (Figure 8a) from where the fibres are likely to be pulled out, indicating poor fibre wetting, unlike other composites in which fibres were concealed by the matrix (Figure 8b,c). The composites made with polymer sheets of 0.56 or 0.29 mm did not show significant differences between their tensile properties, which is thought to be due to the similar levels of fibre wetting within these composites (Figure 8b compared to Figure 8c). However, composites made with polymer sheets of 0.56 mm showed significant decreases in tensile strength and Young’s modulus at the fibre content of 45 wt% due to poor fibre wetting (Figure 9a) as a result of large distances the polymer needs to travel (Table 1 and Table 3).

Composites made with polymer sheets of 0.29 mm showed progressive increases in tensile strength and Young’s modulus with increasing fibre content up to 60 wt%, and thereafter significant decreases. This is thought to be due to the reasonable fibre wetting (Figure 9b,c) throughout these composites as a result of the least distance (the distance between two polymer sheets) the polymer needs to travel. However, it should be noted that the composites with fibre contents above 40 wt% were not fully free of defects; a few gaps between the fibres and the matrix and some voids were visible in the fracture surfaces of these composites. The formation of voids at high fibre content in composites could be due to the evaporation of a greater amount of moisture from the fibres. Similar observations for composites with fibre contents above 40 wt% have been reported elsewhere [21]. Reduction in tensile properties at about fibre content of 70 wt% is thought to be due to insufficient polymer (Table 3) for adequate wetting (Figure 9d). The strongest and stiffest composite contained 60 wt% fibre content and had average tensile strength and Young’s modulus of 44.8 MPa and 6.2 GPa, respectively, which were 2.9 and 6.5 times higher than those of the control.

### 3.3. Flexural Properties of Composites

**Figure 10:**
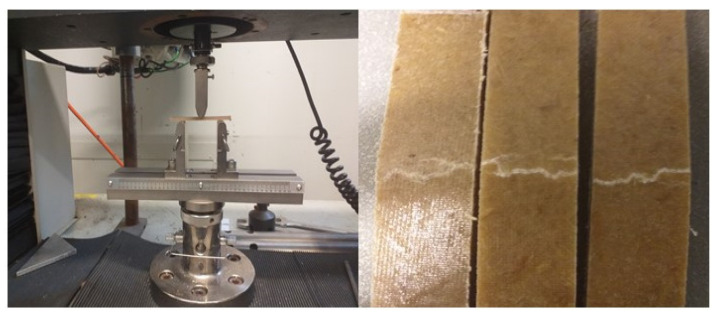
Sample picture of flexural testing samples (composite samples with 30 wt% fibre content) showing the cracks formed from the tensile sides.

Figure 10 shows the tensile side view of the fractured flexural samples. As can be seen, the cracks appeared on the tension surfaces of the samples. It was observed during testing that crack propagated to the middle through the sample thickness with no buckling or delamination in composites.

**Figure 11:**
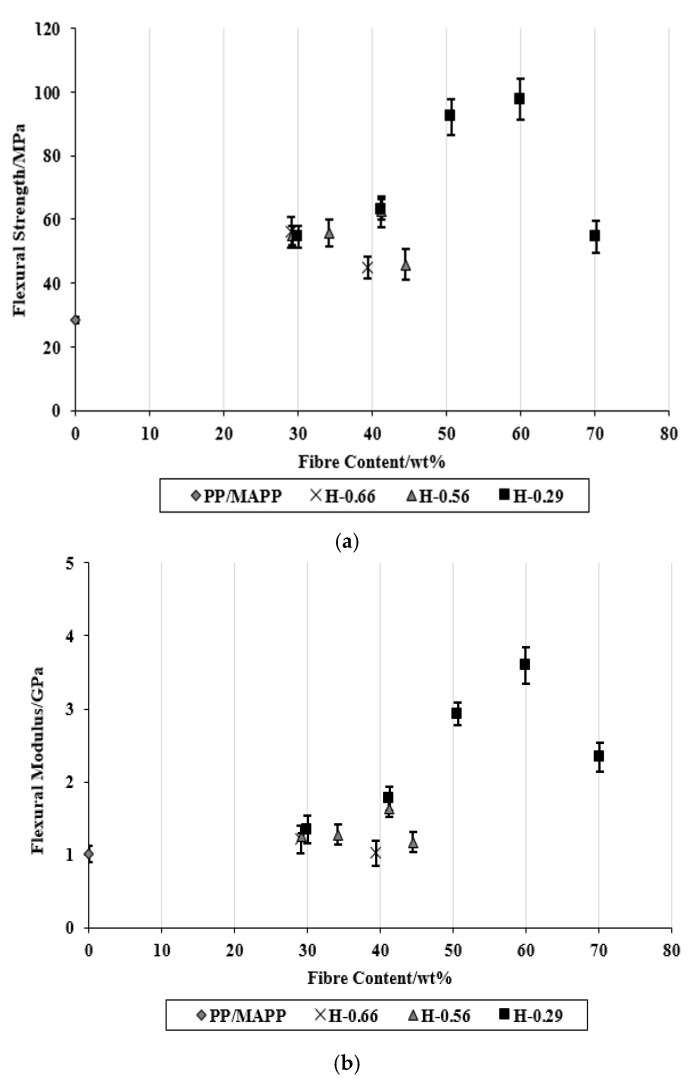
(**a**) Flexural strength and (**b**) flexural modulus of composites as a function of fibre content.

The average flexural strength and flexural modulus of the composites with different fibre contents are presented in Figure 11. The effect of polymer sheet thicknesses and stacking arrangements on flexural properties appeared to be almost similar to that observed for the tensile properties. It has been previously reported that the fracture micro-mechanics that occurs in the composites under flexural testing is similar to tensile testing [7]. The maximum flexural strength and flexural modulus occurred for composites at the fibre content of about 60 wt% were 97.6 MPa and 3.6 GPa, respectively. At this fibre content, flexural strength and flexural modulus were about 244% and 256%, respectively, higher than those of the control.

### 3.4. Impact Strength of Composites

**Figure 12:**
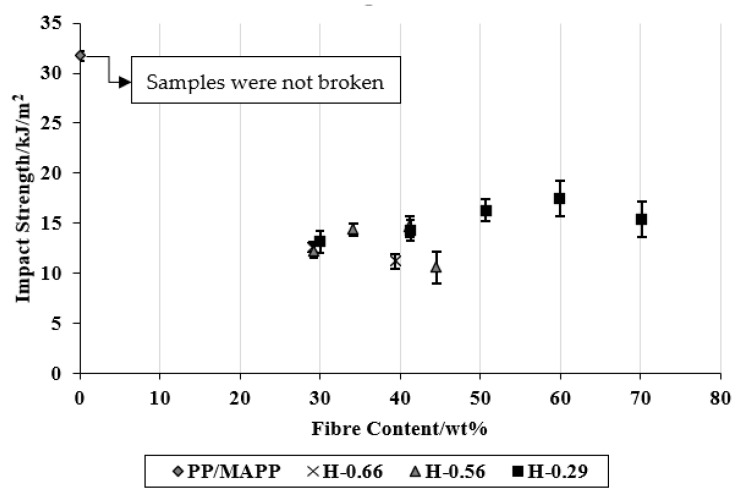
The average impact strength of composites as a function of fibre content.

The average impact strengths of unnotched control and composite samples are shown in Figure 12. It should be noted that the control samples were not broken during the testing. The impact strength of the control samples decreased drastically with the inclusion of fibres. The reduction in impact strength of thermoplastics such as PP with the inclusion of natural fibres is commonly observed in the literature [26]. However, for the composites, the effect of polymer sheet thicknesses and stacking arrangements on impact strength appeared similar to that observed for the tensile and flexural properties. It has been previously reported that the interfacial bonding between the fibre and matrix plays a vital role in determining the mechanical properties of composites [27].

### 3.5. Density and Porosity of Composites

**Figure 13:**
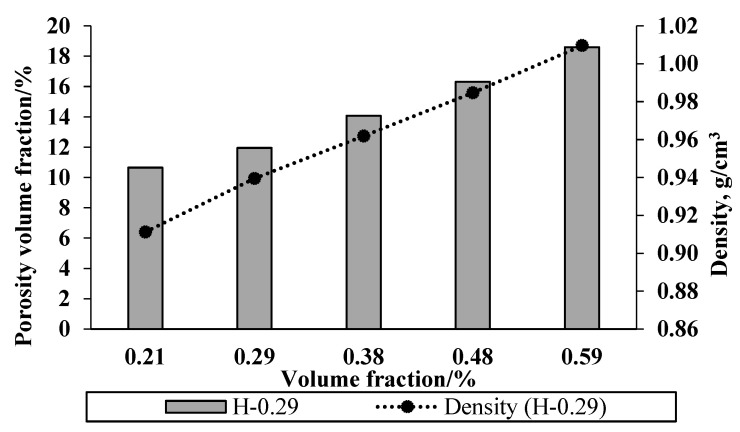
Density and porosity of composites as a function of the volume fraction of fibres.

Both density and porosity of the composites were found to increase with increasing volume fraction of fibres (Figure 13). This is due to the fact that the density of fibre being higher than that of the matrix [28]. Typically, significant porosity has been shown by natural fibre composites and has been shown to increase with increasing fibre content [29]. Porosity arises mainly due to the limited interaction between the fibre and the matrix and inclusion of air during processing.

## 4. Conclusions

Different polymer sheet thicknesses and stacking arrangements were investigated to improve the mechanical performance of the polypropylene matrix composites through achieving high hemp fibre content. It was found that decreasing the overall thickness of fibre mats between two polymer sheets within the stacking arrangements of composites, and so decreasing the distance the polymer needs to travel, improved the fibre wetting through the composite and therefore improved the tensile properties of the composite. The maximum mechanical properties (tensile properties, flexural properties, and impact strength) of the polypropylene composites reinforced with aligned hemp fibre mats were found at a fibre content of 60 wt%. The tensile strength and Young’s modulus of the composites were found to be 44.8 MPa and 6.2 GPa, respectively; the flexural strength and flexural modulus of the composites were found to be 97.6 MPa and 3.6 GPa, respectively and the impact strength of the composites was found to be 18 kJ/m^2^. 

## Figures and Tables

**Table 1:** Abbreviations and stacking arrangements of polymer sheets (PP/MAPP) and hemp fibre mats in the mould.

Samples *	Targeted Fibre wt%	PP* Sheets No.	Fibre Mats No.	Actual Fibre wt%	Stacking Arrangement from Bottom to the Top of the Mould
H30–0.66		3	6	29.1	1PP*/3MATS/1PP*/3MATS/1PP*
H30–0.56		3	6	29.5	1PP*/3MATS/1PP*/3MATS/1PP*
H30–0.29	30	4	6	30.0	1PP*/2MATS/1PP*/2MATS/1PP*/2MATS/1PP*
H30–0.24		4	6	31.8	1PP*/2MATS/1PP*/2MATS/1PP*/2MATS/1PP*
H35–0.56	35	5	8	34.2	1PP*/2MATS/1PP*/2MATS/1PP*/2MATS/1PP*/2MATS/1PP*
H40–0.66		3	8	39.4	1PP*/4MATS/1PP*/4MATS/1PP*
H40–0.56	40	5	10	41.2	1PP*/3MATS/1PP*/2MATS/1PP*/2MATS/1PP*/3MATS/1PP*
H40–0.29		6	10	41.3	1PP*/2MATS/1PP*/2MATS/1PP*/2MATS/1PP*/2MATS/1PP*/2MATS/1PP*
H45–0.56	45	5	12	44.5	1PP*/3MATS/1PP*/3MATS/1PP*/3MATS/1PP*/3MATS/1PP*
H50–0.29	50	8	14	50.1	1PP*/2MATS/1PP*/2MATS/1PP*/2MATS/1PP*/2MATS/1PP*/2MATS/1PP*/2MATS/1PP*/2MATS/1PP*
H60–0.29	60	10	18	59.9	1PP*/2MATS/1PP*/2MATS/1PP*/2MATS/1PP*/2MATS/1PP*/2MATS/1PP*/2MATS/1PP*/2MATS/1PP*/2MATS/1PP*/2MATS/1PP*
H70–0.29	70	12	22	70.2	1PP*/2MATS/1PP*/2MATS/1PP*/2MATS/1PP*/2MATS/1PP*/2MATS/1PP*/2MATS/1PP*/2MATS/1PP*/2MATS/1PP*/2MATS/1PP*/2MATS/1PP*/2MATS/1PP*

Note the following: PP* = PP/MAPP, MATS = hemp fibre mats produced using DSF. * In the abbreviation ‘H’ refers to hemp composites, the number following ‘H’ refers to the nominal weight percentage (wt%) of fibres and the final number refers to the thickness (in mm) of the polymer sheets used in the production of composites.

**Table 2:** The polymer sheet production processes and the overall average thickness of the polymer sheets. Standard deviations are shown in parentheses.

Sheet Production Processes	Hot Press Temperature/°C	Overall Average Sheet Thickness/mm
Extrusion using die spacer of 1.00 mm	-	0.66 (0.05)
Extrusion using die spacer of 0.50 mm	-	0.56 (0.06)
Extrusion using die spacer of 0.50 mm + Pressing between the plates in a hot press	(a) 130 °C (b) 140 °C (c) 150 °C (d) 160 °C	(a) 0.49 (0.03) (b) 0.29 (0.04) (c) 0.24 (0.02) (d) 0.13 (0.06)

**Table 3:** Ratio of thicknesses of polymer sheets to fibre mats in composites.

Samples *	Average Thickness of a Fibre Mat/mm	Total Thickness of Fibre Mats in Composites/mm	Total Polymer Sheet Thicknesses at Edges in Composites/mm	Total Polymer Sheet Thicknesses at Middle in Composites	Ratio of Thicknesses of Polymer Sheets to Fibre Mats at Edges in Composites	Ratio of Thicknesses of Polymer Sheets to Fibre Mats at Middle in Composites
H30–0.66	0.80	4.8	1.83	2.40	0.381	0.500
H30–0.56		4.8	1.56	1.89	0.325	0.394
H30–0.29		4.8	1.08	1.32	0.225	0.275
H30–0.24		4.8	0.84	1.08	0.175	0.225
H35–0.56		6.4	2.60	3.15	0.406	0.492
H40–0.66		6.4	1.83	2.40	0.286	0.375
H40–0.56		8.0	2.60	3.15	0.325	0.394
H40–0.29		8.0	1.62	1.98	0.203	0.248
H45–0.56		9.6	2.60	3.15	0.271	0.328
H50–0.29		11.2	2.16	2.64	0.193	0.236
H60–0.29		14.4	2.70	3.30	0.188	0.229
H70–0.29		17.6	3.24	3.96	0.184	0.225

* In the abbreviation ‘H’ refers to hemp composites, the number following ‘H’ refers to the nominal weight percentage (wt%) of fibres and the final number refers to the thickness (in mm) of the polymer sheets used in the production of composites.

## Data Availability

The data presented in this study are provided upon request.
